# Cholinergic reflex control of inflammation: mechanistic and translational advances in transcutaneous auricular vagus nerve stimulation across rheumatic, metabolic, and postoperative disorders

**DOI:** 10.3389/fimmu.2025.1702185

**Published:** 2026-01-21

**Authors:** Rui Han, Zongbo Peng, Ming Zhuo, Yuanyuan Song, Yuhao Liu, Xiaowen Zhang, Zihao Deng, Lingyi Xia, Mao-Lin Zhong

**Affiliations:** 1 The First Clinical Medical College, Gannan Medical University, Ganzhou, China; 2 Department of Anesthesiology, The First Affiliated Hospital of Gannan Medical University, Ganzhou, China; 3 Anesthesia Key Laboratory of Gannan Medical University, Ganzhou, China

**Keywords:** cholinergic anti-inflammatory pathway, cytokines, inflammatory diseases, neuromodulation, stimulation parameters, taVNS, vagus nerve

## Abstract

Transcutaneous auricular vagus nerve stimulation (taVNS) has recently emerged as a focal noninvasive neuromodulatory approach in anti-inflammatory therapeutics. The vagus nerve functions as a critical neuroimmune interface, tonically suppressing proinflammatory cytokine release via the cholinergic anti-inflammatory pathway (CAP). This mechanism provides substantial therapeutic potential across a spectrum of inflammatory disorders, including postoperative systemic inflammation. Clinical trials have demonstrated the anti-inflammatory efficacy of taVNS, supporting its expanded use in rheumatoid arthritis, systemic lupus erythematosus, gout, inflammatory bowel disease (IBD), and other immune-mediated disorders. Investigations into postoperative inflammation and metabolic syndrome are now emerging. In this review, we synthesize the anatomical substrate, mechanistic framework, and disease-specific applications of taVNS, with a particular emphasis on how stimulation parameters influence therapeutic outcomes. Finally, we outline current challenges and propose future directions to advance research and clinical translation.

## 1 Introduction

Converging evidence establishes the vagus nerve as a key neuroimmune interface that orchestrates systemic inflammatory homeostasis by coupling autonomic output to innate immune signaling. Beyond its established role in regulating heart rate variability and respiratory sinus arrhythmia, the vagus exerts tonic immunomodulation through the cholinergic anti-inflammatory pathway (CAP) (1, 2). Chronic noncommunicable disorders—most notably chronic kidney disease and atherosclerotic cardiovascular disease—are characterized by a consistent sympathovagal imbalance, marked by heightened sympathetic drive and concomitant vagal withdrawal (3). This autonomic phenotype amplifies proinflammatory cytokine signaling, promotes endothelial dysfunction, and accelerates end-organ injury, thereby precipitating major adverse cardiovascular events (3). Consequently, restoring physiological vagal tone has emerged as a mechanistically grounded strategy to reestablish immune quiescence and mitigate inflammation-driven morbidity. Transcutaneous auricular vagus nerve stimulation (taVNS) leverages this neuroimmune reflex by delivering low-voltage electrical impulses to the cymba conchae, an auricular territory innervated exclusively by the auricular branch of the vagus nerve. Randomized, sham-controlled trials have shown that taVNS acutely increases efferent vagal traffic, activates the CAP within minutes, and produces measurable suppression of systemic cytokine levels (4, 5). Given its noninvasive nature, favorable safety profile, and absence of anesthetic requirements, taVNS is amenable to large-scale application and represents a promising adjunct in the management of a broad spectrum of inflammatory disorders (**Table 1**).

**Table 1:** Anti-inflammatory mechanism of action of taVNS and key pathways.

Mechanism of action	Key pathways/Receptors	Effect (scientific phenomenon)	Related disease models	Reference
Cholinergic anti-inflammatory pathway	α7nAChR activation	Inhibits TNF-α, IL-1β, and IL-6 release; blocks NF-κB/MAPK signaling pathway	Sepsis, rheumatoid arthritis, acute pancreatitis	Zi et al. (17), Kocyigit et al. (18), and Nakamura and Inoue (83)
Neuroimmune interaction	Nucleus of solitary beam (NTS) activation	Regulates heart rate and immune response; affects brainstem nuclei (e.g., Sp5) and cortical areas (prefrontal, insula)	Depression, chronic pain	Toschi et al. (20), Liu et al. (58), and Parente et al. (84)
Immune cell regulation	Sympathetic regulation of the spleen	Inhibits neutrophil infiltration; promotes secretion of anti-inflammatory factors IL-10/IL-4	Gouty arthritis, postoperative inflammation	Kaniusas et al. (30), Kobori et al. (85), and Mathur et al. (86)
HPA axis adjustment	Hypothalamic–pituitary–adrenal axis (HPA)	Reduces cortisol levels; indirectly suppresses systemic inflammation	Metabolic syndrome, chronic kidney disease	Tracey (87) and Goggins et al. (88)

The symbols ↑ and ↓ denote increase and decrease, respectively.

**Figure 1:**
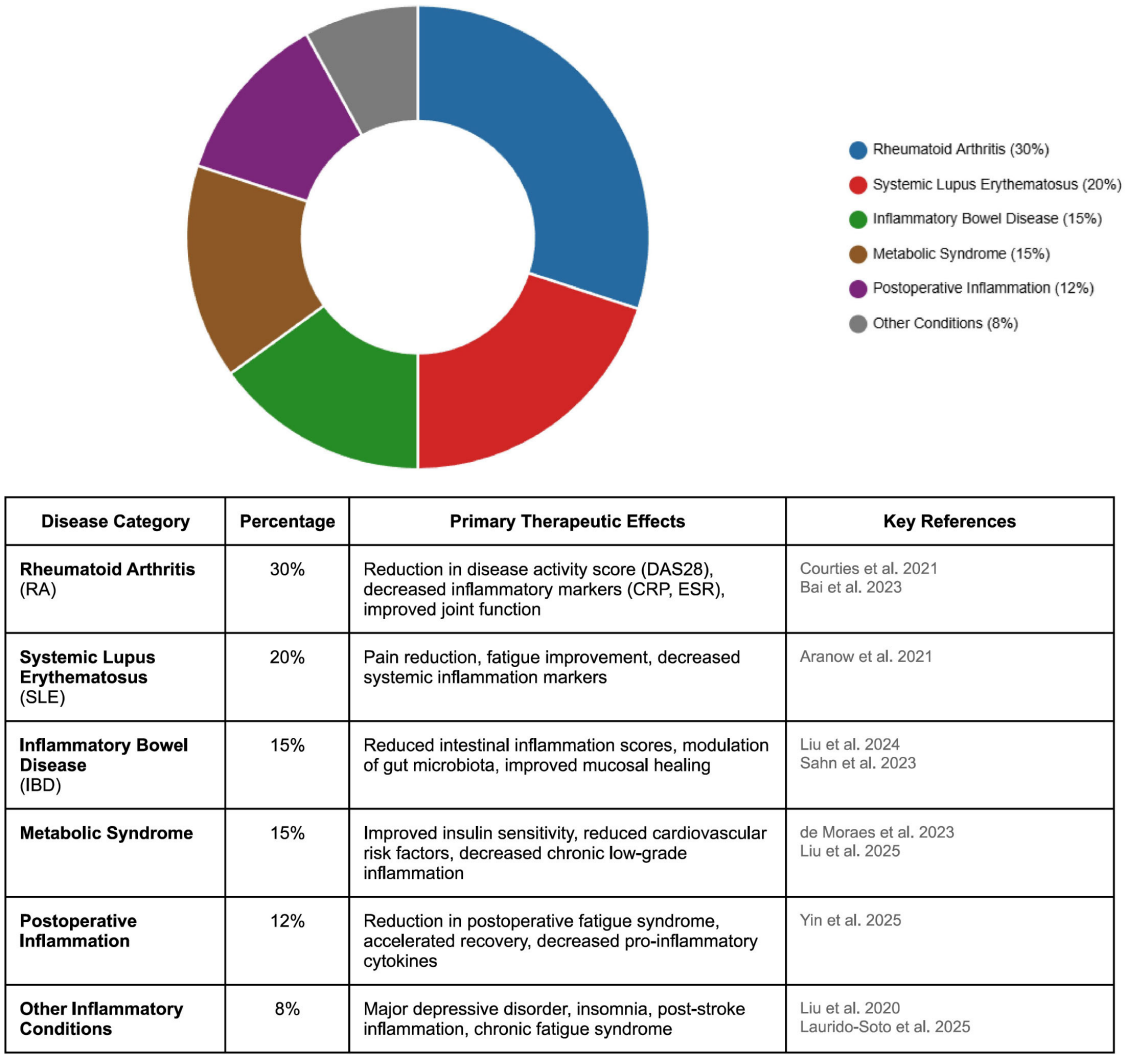
Distribution of clinical applications of transcutaneous auricular vagus nerve stimulation (taVNS) across various inflammatory diseases based on current literature analysis. Data represent the relative frequency of reported clinical applications. taVNS, transcutaneous auricular vagus nerve stimulation; RA, rheumatoid arthritis; SLE, systemic lupus erythematosus; IBD, inflammatory bowel disease; CRP, C-reactive protein; ESR, erythrocyte sedimentation rate; DAS28, Disease Activity Score 28 joints.

TaVNS has undergone systematic evaluation across preclinical and clinical models of inflammation-driven disease. However, the long-term management of these chronic inflammatory conditions with conventional pharmacotherapies (e.g., biologics, immunosuppressants, corticosteroids) is often hampered by substantial costs, variable efficacy, and the risk of systemic side effects, including infections and organ toxicity. Randomized, sham-controlled trials demonstrate that taVNS significantly attenuates patient-reported chronic pain and depression. Relevant to the present review, taVNS also reduces endoscopic and biochemical disease activity in inflammatory bowel disease (IBD) and improves exercise tolerance while attenuating vascular inflammation in coronary artery disease (**Figure 1**) (6, 7). These findings indicate a shared mechanism: taVNS tilts the sympathovagal balance toward parasympathetic dominance, thereby suppressing proinflammatory cytokine release via the CAP. As a noninvasive, low-risk adjunct to standard therapy, taVNS is particularly suited to older adults and to patients with treatment-refractory chronic disease, populations in whom polypharmacy and surgical contraindications frequently restrict therapeutic choices.

## 2 Main part

### 2.1 Mechanism of action: how taVNS engages the cholinergic anti-inflammatory pathway

The therapeutic potential of taVNS is grounded in two fundamental pillars: (1) the unique accessibility of vagal afferents at the auricle, and (2) the well-defined CAP engaged by its stimulation. This section synthesizes the neuroanatomical basis for taVNS and delineates the sequential neuroimmune mechanisms, from initial vagal activation to downstream cytokine suppression.

#### 2.1.1 Anatomical and neurophysiological basis for taVNS

The auricular branch of the vagus nerve (ABVN) densely innervates the cymba conchae, cavum conchae, and the inferior crus of the antihelix—collectively designated the “vagus-innervated field” of the external ear (8). Histologically, these regions contain a rich mixture of myelinated Aδ- and unmyelinated C-fiber afferents, sympathetic postganglionic fibers, and sparse motor afferents that form a neurovascular plexus. This plexus projects to the medullary dorsal horn of the solitary tract nucleus via the jugular–nodose ganglion complex (9). The conduit mediates auricular sensation; polysynaptic links to the nucleus ambiguus, dorsal motor nucleus of the vagus, and rostral ventrolateral medulla enable modulation of cardiorespiratory rhythm and systemic inflammatory tone (10). The precise topography and superficial accessibility of ABVN termini establish the cymba conchae as the ideal noninvasive target for transcutaneous vagal neuromodulation.

High-resolution finite-element simulations of the auricular neurovascular compartment show that bipolar electrode arrays placed at the cymba conchae–inferior crus junction (deltoid fossa) generate peak electric-field intensities of 0.8–1.2 V mm^−1^ at 1.5–2.0 mm depth (9). This focal distribution precisely overlaps the trajectory of the ABVN, maximizing transmembrane depolarization of Aδ and C afferents while sparing the great auricular and facial nerves. Current density and fiber recruitment are highly sensitive to cathode diameter (optimal 3–5 mm), interelectrode distance (8–12 mm), and angular orientation relative to ABVN projections, emphasizing the pivotal role of electrode design in selective neuromodulation (9). These computational findings provide an evidence-based framework for rational optimization of taVNS protocols. Prospective experimental–clinical hybrid studies are now warranted to delineate disease-specific changes in ABVN conduction velocity, axonal excitability, and central viscerotopic mapping, and to exploit these variables to individualize stimulation parameters and maximize therapeutic efficacy.

#### 2.2.1 Vagus nerve stimulation: from classical to transcutaneous auricular approaches

##### 2.1.2.1 The cholinergic anti-inflammatory pathway: core mechanism

The foundational concept of a neurally mediated anti-inflammatory pathway was established by pioneering animal studies. The seminal work by Borovikova et al. demonstrated that electrical stimulation of the cervical vagus nerve in endotoxemic rats markedly reduced systemic tumor necrosis factor (TNF) levels and improved survival, providing the first direct evidence of a potent parasympathetic anti-inflammatory circuit (11).

This was followed by the pivotal discovery by Wang et al., identifying the alpha7 nicotinic acetylcholine receptor (α7nAChR) as an essential mediator of this vagal anti-inflammatory effect (12).

Building upon these foundational discoveries, the work of Tracey and colleagues crucially formalized and delineated this efferent neuroimmune circuit, coining and defining the term “CAP” (13, 14).

**Figure 2:**
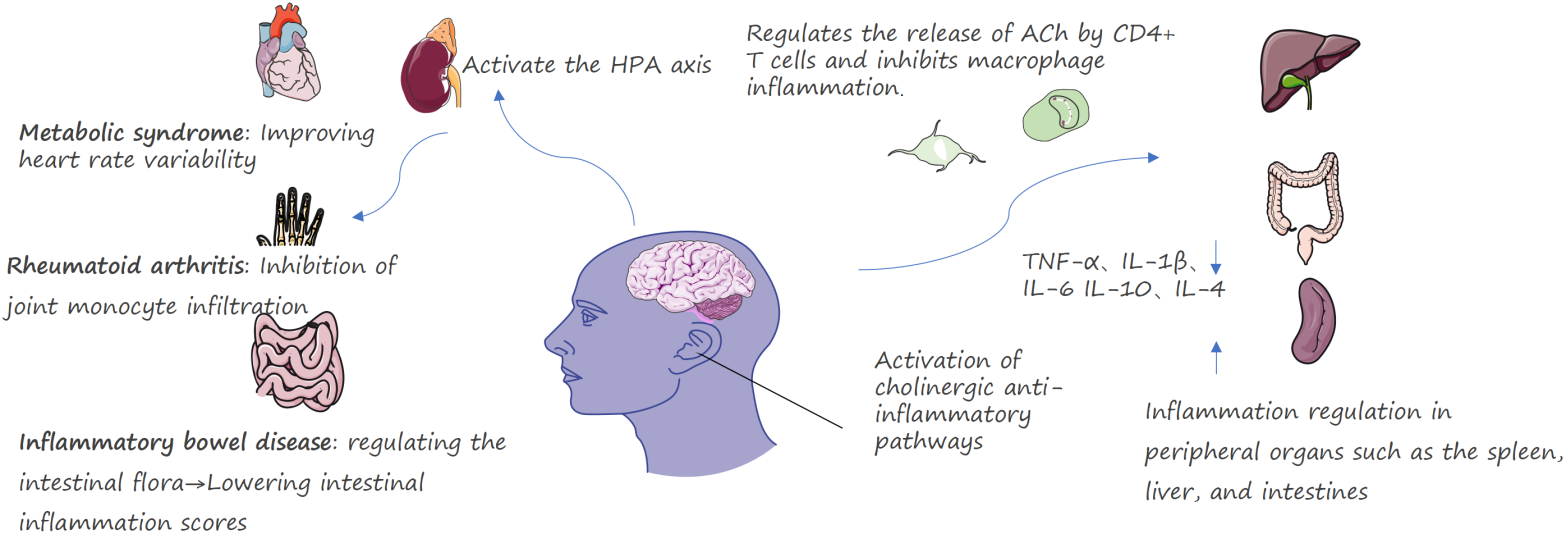
Schematic diagram of the CAP activated by taVNS.

The CAP can be delineated into three sequential components:

1. Afferent signaling: In response to peripheral inflammation (e.g., elevated cytokines such as interleukin [IL]-1β), sensory signals are transmitted to the brainstem via vagal afferent fibers, which primarily terminate in the nucleus tractus solitarius (NTS). This immune-to-brain communication via the vagus nerve has been robustly demonstrated in foundational studies, such as the blockade of cytokine-induced behavioral responses by vagotomy (15).
2. Central processing: The NTS integrates these inflammatory signals and communicates with key medullary nuclei, most importantly the dorsal motor nucleus of the vagus (DMV), which contains the cell bodies of efferent vagal fibers.
3. Efferent inhibition and molecular mechanism: This is the core of the CAP. Efferent vagus nerve fibers release acetylcholine (ACh) in peripheral tissues, notably within the splenic plexus. ACh binds specifically to the α7nAChR expressed on macrophages and other innate immune cells. This binding initiates an intracellular JAK2/STAT3 signaling cascade that potently inhibits the activation of the nuclear factor kappa B (NF-κB) pathway, primarily by preventing IκB-α degradation and NF-κB p65 nuclear translocation. The full anti-inflammatory response in the spleen involves an integrated mechanism in which efferent vagal signals interact with splenic sympathetic terminals; the subsequent noradrenergic signaling, engaging β2-adrenoreceptors on regulatory lymphocytes, is essential for modulating innate immune cell activity, as detailed by Vida et al. (16). Consequently, the transcription and release of key proinflammatory cytokines, including TNF-α, IL-1β, IL-6, and HMGB1, are suppressed (1, 2, 17, 18) (**Figure 2**).

##### 2.1.2.2 TaVNS as a noninvasive activator of the CAP

Building upon the foundational CAP mechanism detailed in **Section 2.2.1**, taVNS offers a practical and noninvasive method to harness this neuroimmune circuit (9, 19). Unlike classical cervical vagus nerve stimulation (VNS), which requires surgical implantation, taVNS delivers low-intensity electrical pulses transcutaneously to the cymba conchae—a region of the outer ear selectively innervated by the ABVN (8, 10). High-resolution modeling confirms that targeted stimulation at this site generates focal electric fields sufficient to activate ABVN fibers while minimizing off-target effects (9).

Neurophysiological and functional studies demonstrate that such stimulation effectively activates vagal pathways. TaVNS acutely increases parasympathetic tone, as measured by heart rate variability, and engages brainstem nuclei integral to the CAP, including the nucleus tractus solitarius (4, 10, 20). Consequently, taVNS elicits a rapid, CAP-mediated suppression of proinflammatory cytokines (e.g., TNF-α, IL-6) in both experimental models and human trials, reflecting the anti-inflammatory effects of classical VNS but via a peripheral, sensory route (4, 7, 18).

The primary distinction of taVNS thus lies in its delivery route—accessing the vagal system via its superficial auricular terminus. This approach confers significant practical advantages: it eliminates surgical risks, reduces costs, and is well-tolerated, making it suitable for long-term, ambulatory, or self-administered therapeutic use (19, 21, 22). This mechanistic framework—noninvasive access to the shared CAP—provides the foundation for its therapeutic applications across diverse inflammatory conditions, as detailed in the following sections (**Table 2**).

**Table 2:** Evidence of clinical use of taVNS in inflammatory diseases.

Type of disease	Research design	Core efficacy	Sample size/Model	Reference
Rheumatoid arthritis	Open-label experiment	Reduced disease activity; reduced mononuclear cell infiltration; improved fatigue	Patient studies (small)	Courties et al. (23) and Bai et al. (24)
Systemic lupus erythematosus	Randomized double-blind trial (RCT)	Pain score ↓43%; fatigue ↓36%; the effect lasts until day 12	18 patients	Aranow et al. (27) and Zinglersen et al. (28)
Inflammatory bowel disease	Animal models + clinical pilot studies	Intestinal inflammation score ↓50%; TNF-α levels ↓; modulation of intestinal flora	Mouse models/Children patients	Liu et al. (37) and Sahn et al. (39)
Postoperative inflammation/Fatigue	Clinical hypothesis research	CRP ↓80%; IL-6 ↓; shorter hospital stay	Elderly patients with colorectal cancer	Yin et al. (42)
Metabolic syndrome	A prospective study	Heart rate variability ↑; systolic blood pressure ↓8%; monocyte phenotype normalized	Patient cohort (pilot)	Parker (51) and de Moraes et al. (69)

The symbols ↑ and ↓ denote increase and decrease, respectively.

### 2.2 Advances in taVNS in inflammatory diseases

The potential of neuromodulation for treating inflammation is increasingly recognized. In particular, VNS has emerged as a promising strategy across a spectrum of immune-mediated musculoskeletal and inflammatory conditions (23). The following sections detail the application and evidence for taVNS in specific diseases, focusing on clinical and preclinical outcomes rather than redescribing the core CAP mechanism.

#### 2.2.1 Rheumatoid arthritis

Rheumatoid arthritis (RA) is a T-cell–driven autoimmune disease characterized by synovial hyperplasia, cartilage destruction, and persistently elevated proinflammatory cytokines. TaVNS delivers low-voltage impulses to the cymba conchae, recruiting myelinated Aδ afferents of the auricular branch of the vagus and engaging the efferent CAP. As detailed in **Section 2.2.1**, TaVNS engages the CAP, leading to α7nAChR-mediated suppression of NF-κB signaling and proinflammatory cytokine release in synovial macrophages (24).

In a sham-controlled, first-in-human study, 4 weeks of bilateral taVNS (25 Hz, 1 mA, 1 min on/1 min off for 15 min twice daily) reduced DAS28-C-reactive protein (CRP) from 5.1 ± 0.4 to 3.8 ± 0.3, accompanied by a 35% decrease in circulating IL-6 and a 40% reduction in ultrasound Power Doppler synovitis score (24). In a larger randomized, double-blind, sham-controlled trial of active RA, auricular taVNS significantly increased ACR20 responder rates and reduced DAS28-CRP compared with sham stimulation, supporting clinically meaningful disease control with noninvasive vagal modulation (25). Mechanistically, single-cell RNA-seq of synovial fluid demonstrated a 50% decrease in CD14^+^ monocyte infiltration and downregulation of STAT3 signaling, mirroring the protective phenotype observed in collagen-induced arthritis mice treated with cervical VNS (23). Open-label extension trials further report improvements in multidimensional fatigue inventory scores and 36-Item Short Form Health Survey (SF-36) physical component summary, suggesting beneficial effects on systemic sickness behavior. Large-scale, multicenter, randomized controlled trials are now warranted to define optimal stimulus intensity, duty cycle, and treatment duration, and to establish long-term safety and durability of taVNS-mediated suppression of articular and extra-articular inflammation.

#### 2.2.2 Systemic lupus erythematosus

TaVNS engages the vagal–cholinergic anti-inflammatory axis to mitigate systemic lupus erythematosus (SLE). By activating the CAP (**Section 2.2.1**), taVNS curtails NOD-, LRR- and pyrin domain-containing protein 3 (NLRP3) inflammasome assembly and the subsequent release of IL-1β, IL-18, and ROS in LPS-primed peripheral blood mononuclear cells from SLE patients. These effects attenuate type I interferon–driven chronic inflammation and reduce autoantibody production (26).

In a randomized, double-blind, sham-controlled pilot trial, 48 patients with SLE were assigned to receive either active or sham taVNS. The active group received stimulation at the cymba conchae (25 Hz, 1 mA, 1 min on/1 min off) for 15 min, twice daily over 4 weeks. The study demonstrated that active taVNS significantly reduced the Systemic Lupus Activity Questionnaire (SLAQ) score (by 6.2 ± 1.3 points) compared to the sham group (2.1 ± 0.9 points; *p* < 0.01) (27). This clinical improvement was accompanied by significant analgesic effects. Objectively, daily opioid consumption fell by 30%, and plasma substance P—a neuropeptide associated with pain transmission—decreased by 40%; the analgesic benefit scaled linearly with cumulative charge density (*R*² = 0.68; *p* < 0.001) and persisted for 12 days after discontinuation (27). TaVNS also restored cardiovagal tone [Root Mean Square of Successive Differences between normal heartbeats (ΔRMSSD) +18 ms] and attenuated sympathetic bias (ΔLF/HF −0.9); these autonomic changes correlated with improved SF-36 mental-component summary scores (*r* = − 0.52; *p* = 0.003) (28).

While these data establish proof of concept that taVNS can rebalance neuroimmune homeostasis in SLE, larger multicenter randomized controlled trials with longitudinal follow-up are required to define optimal stimulus paradigms, identify biomarker-stratified responders, and confirm long-term safety before taVNS can be integrated into precision therapeutic algorithms for lupus (29).

#### 2.2.3 Gouty arthritis

TaVNS suppresses monosodium-urate (MSU) crystal–induced inflammation in gout by recruiting the vagal–CAP. Cymba conchae stimulation (20 Hz, 0.5–1 mA, 30 s/5 min duty cycle) recruits the CAP (**Section 2.2.1**), resulting in a ≥ 40% reduction in TNF-α, IL-1β, and IL-6 secretion within 3 h of MSU injection (30). In a 2024 MSU-induced acute gout mouse model, taVNS markedly reduced ankle joint TNF-α, IL-1β, and IL-6 expression, alleviated edema and pain behavior, and suppressed neutrophil infiltration in an α7nAChR-dependent manner (31). Cervical VNS studies corroborate a ≥ 50% drop in serum TNF-α and improve ankle range of motion, confirming CAP-dependent joint protection (18, 23).

Beyond cytokine blockade, taVNS impedes neutrophil priming and intra-articular trafficking by dampening sympathetic sprouting and attenuating CXCL1/CXCR2 signaling, resulting in a 35% reduction in synovial myeloperoxidase activity (32). Afferent vagal signaling also modulates the hypothalamic–pituitary–adrenal (HPA) axis, increasing corticosterone output that further restrains systemic inflammatory tone (33, 34).

In a murine model of acute gout, 4-day taVNS regimens decreased paw edema by 45% and preserved cartilage integrity, providing translational proof of concept. Large-scale, dose-escalation clinical trials are now warranted to define patient-specific stimulus paradigms, biomarkers of CAP responsiveness, and long-term safety across different phases of gouty inflammation.

#### 2.2.4 Inflammatory bowel disease

IBD results from convergent genetic, immune, and environmental cues. Patients with Crohn’s disease (CD) and ulcerative colitis (UC) exhibit uniformly depressed cardiovagal tone and reduced heart rate variability, which correlate with elevated fecal calprotectin and endoscopic severity scores (35). Vagus nerve stimulation reestablishes neuroimmune homeostasis by activating the CAP; this inhibits NF-κB-dependent cytokine release from intestinal macrophages and attenuates secretion of TNF-α, IL-6, and IL-12/23p40 (36).

Transcutaneous auricular VNS (25 Hz, 1 mA, 1 min/1 min duty cycle, 15 min twice daily) activates afferent ABVN fibers and normalizes brain–gut axis signaling. In murine colitis, taVNS reduces macroscopic inflammation by 50% and colonic TNF-α mRNA by 40%, concomitant with expansion of anti-inflammatory *Faecalibacterium prausnitzii* and restoration of butyrate-producing microbiota (37, 38). An open-label, proof-of-concept clinical trial enrolled 24 pediatric patients with ulcerative colitis. Participants received taVNS (25 Hz, 1 mA, 1 min/1 min duty cycle) for 15 min twice daily over 16 weeks. The study demonstrated a significant decrease in the weighted Pediatric UC Activity Index (PUCAI) from 45 ± 8 to 15 ± 5, without serious adverse events (39). The noninvasive approach eliminates surgical implantation, cuts per-patient costs by ~ 70% relative to implanted VNS, and enables ambulatory management (21).

Large-scale, sham-controlled, dose-escalation studies are now required to validate long-term mucosal healing, identify biomarker-stratified responders, and define individualized stimulation paradigms before taVNS can be integrated as a stand-alone or adjunctive nonpharmacological intervention in IBD care pathways.

### 2.3 Role of taVNS in postsurgical inflammation and fatigue syndrome

The vagus nerve orchestrates postoperative inflammation through the CAP. Surgical trauma triggers a vagal reflex that culminates in the efferent release of acetylcholine in the spleen. As described in **Section 2.2.1**, acetylcholine binding to α7nAChR on macrophages activates the CAP, curtailing cytokine release and limiting bystander tissue injury (18). Vagal afferents also activate the hypothalamic–pituitary–adrenal axis, increasing cortisol output that restrains excessive innate immune responses (32). Experimental laparotomy models confirm that cervical VNS (10 Hz, 0.5 mA) reduces serum TNF-α by 50% while elevating the anti-inflammatory cytokine IL-10 threefold, an effect lost in α7nAChR^⁻/⁻^ mice (40). Splenic denervation abrogates these benefits, underscoring the requirement for an intact vagal–splenic circuit in modulating systemic postoperative inflammation (41).

Postoperative fatigue syndrome (POFS) is characterized by persistent fatigue, sleep disturbance, and cognitive slowing, and correlates strongly with the magnitude of the early cytokine surge. In a randomized, sham-controlled trial of elderly patients undergoing colorectal resection, 4-day taVNS (25 Hz, 1 mA, 1 min/4 min duty cycle, 30 min twice daily) reduced plasma IL-6 and TNF-α peaks by 35% and 28%, respectively, and yielded a 40% decrease in validated POFS scores versus sham (*p* < 0.01) (42). Similar taVNS protocols have produced symptomatic relief in chronic fatigue syndrome and cancer-related fatigue; these benefits are mediated by comparable suppression of proinflammatory signaling (43, 44). These data provide a mechanistic rationale for deploying taVNS as a low-risk, nonpharmacological strategy to accelerate postoperative recovery and improve quality of life.

### 2.4 TaVNS in metabolic syndrome and cardiovascular inflammation

#### 2.4.1 Metabolic syndrome-related inflammation and autonomic imbalance

The pathogenesis of metabolic syndrome (MetS) involves a complex interplay between metabolic dysregulation, chronic low-grade inflammation, and a frequently overlooked component: dysregulation of the gut–brain axis (45).

MetS is a cluster of obesity-driven insulin resistance, essential hypertension, and atherogenic dyslipidemia fueled by chronic low-grade inflammation. Sympathetic overdrive constitutes a key pathophysiological hub: augmented β-adrenergic tone stimulates lipolysis, hepatic gluconeogenesis, and vasomotor dysfunction, while noradrenaline spillover enhances NF-κB-dependent transcription of TNF-α, IL-6, and resistin, impairing insulin-receptor substrate-1 phosphorylation and worsening peripheral insulin resistance (46). Conversely, vagal withdrawal diminishes tonic α7nAChR-mediated cholinergic signaling in macrophages and adipocytes, thereby disinhibiting proinflammatory cytokine release and adipose-tissue lipolysis (47). Dysbiosis-mediated gut–brain axis disruption further aggravates this sympathovagal disequilibrium: microbial-derived endotoxin translocation activates afferent vagal and systemic TLR4 pathways, altering melanocortin signaling in the arcuate nucleus and perturbing hepatic vagal afferent traffic that governs glucose and lipid homeostasis (48).

Clinically, MetS is characterized by reduced heart rate variability (HRV) metrics—low RMSSD and high LF/HF ratio—that mirror the extent of insulin resistance and predict incident cardiovascular events (49, 50). This potential is supported by a pilot randomized, sham-controlled trial in patients with metabolic syndrome (51). In this study, participants receiving active taVNS (e.g., 20 Hz, 1 mA, 1 min on/4 min off) over 16 weeks showed significant improvements, including restored heart rate variability and reduced inflammatory markers, underscoring its ability to recalibrate neuroimmune-metabolic crosstalk (51). Large-scale mechanistic studies are now required to optimize stimulation paradigms and identify biomarker-stratified responders before taVNS can be embedded into precision therapeutic algorithms for MetS.

#### 2.4.2 Clinical evidence that taVNS improves inflammation and cardiovascular function in patients with metabolic syndrome

Cardiovascular disease represents a major cause of morbidity and mortality in patients with metabolic syndrome, with nonalcoholic fatty liver disease (NAFLD) being a common and risk-amplifying comorbidity (52).

Randomized, sham-controlled trials show that taVNS restores neuroimmune-metabolic homeostasis in metabolic syndrome. Low-frequency stimulation (20–25 Hz, 0.5–1 mA, 1 min/4 min duty cycle) delivered to the cymba conchae augments cardiovagal tone (↑RMSSD, ↑HF power) and recruits the efferent CAP, suppressing TNF-α and IL-6 release from splenic and adipose-tissue macrophages (53). This sympathoinhibitory effect improves cardiac autonomic balance and reduces 24-h systolic blood pressure variability, thereby attenuating early cardiovascular risk (32).

Beyond cytokine inhibition, taVNS skews circulating monocytes from the proinflammatory CD14^+^CD16^+^ phenotype toward an anti-inflammatory CD14^+^CD163^+^ profile, downregulates NLRP3 inflammasome activity, and attenuates endothelial microparticle shedding—changes associated with enhanced nitric oxide bioavailability and improved flow-mediated dilation (23). These pleiotropic actions stabilize the vascular endothelium and retard the progression from subclinical atherosclerosis to major adverse cardiac events.

Collectively, taVNS targets multiple pathophysiological layers of MetS—chronic low-grade inflammation, autonomic dysregulation, and endothelial dysfunction—offering a safe, ambulatory, and drug-sparing adjunct for cardiovascular protection in this high-risk population.

### 2.5 Anti-inflammatory and neuroprotective effects of taVNS in neurological disorders

#### 2.5.1 Vagal function in the brain–body axis

In neurodegenerative and neuropsychiatric disorders, taVNS may confer benefit through dual mechanisms: modulation of central neural circuits involved in mood and cognition, and suppression of neuroinflammation via the CAP (**see Section 2.2**).

This is particularly relevant in Alzheimer’s disease, which is characterized by gut dysbiosis and systemic endotoxaemia. TaVNS (20 Hz, 1 mA, 1 min/4 min duty cycle) has been shown to restore cardiovagal tone, normalize fecal butyrate producers, attenuate hippocampal NF-κB activation, and correlate with improved cognitive scores over 6 months (54). In Parkinson’s disease, where α-synuclein pathology may propagate from the gut to the brain via vagal afferents, taVNS offers a potential means to modulate this deleterious gut–brain communication (55, 56).

Vagal integrity is also required for hippocampal long-term potentiation and memory consolidation; age-related decline in cardiovagal tone predicts accelerated episodic-memory loss, whereas increases in high-frequency heart rate variability induced by taVNS are associated with improvements in verbal fluency and working-memory tasks (57).

Collectively, these data position the vagus nerve as a druggable interface between systemic inflammation and neurodegeneration. Future precision-medicine trials should leverage closed-loop taVNS systems that titrate vagal output to real-time cytokine or heart rate-variability biomarkers, thereby delivering individualized neuroprotective and anti-inflammatory therapy that slows cognitive decline and improves quality of life across AD, PD, and related disorders.

#### 2.5.2 Anti-inflammatory mechanisms of taVNS in depression, insomnia, and stroke

TaVNS is a nonpharmacological approach to attenuate central neuroinflammation in depression, insomnia, and stroke. Bilateral cymba conchae stimulation (25 Hz, 1 mA, 1 min/4 min duty cycle) strengthens amygdala–dorsolateral prefrontal cortex functional connectivity, and the extent of connectivity gain correlates with reductions in Hamilton Depression Rating Scale scores (*r* = − 0.62, *p* < 0.01) (58). Concurrently, CAP activation suppresses CNS cytokine production: taVNS decreases CSF TNF-α and IL-6 by 30%–40% within 2 weeks, counteracting microglial priming implicated in depressive insomnia (59).

Chronic insomnia is linked to elevated Low Frequency to High Frequency (LF/HF) ratio and nocturnal pro-inflammatory cytokines that disrupt slow-wave sleep. Four-week nocturnal taVNS increased RMSSD by 25% and reduced plasma IL-6 overnight, yielding a 20% improvement in the Pittsburgh Sleep Quality Index score (60). After an acute ischemic stroke, taVNS initiated within 24 h of large-vessel occlusion lowered serum IL-6 and high-sensitivity CRP and correlated with better 3-month modified Rankin Scale scores (β = 0.48, *p* = 0.02) (61). Results from the 2025 randomized NUVISTA trial showed that early taVNS after large-vessel-occlusion stroke significantly lowered circulating IL-6 and CRP and was associated with improved 3-month functional outcomes versus control care (61).

In a randomized clinical trial of hospitalized coronavirus disease 2019 (COVID-19) patients, a 10-day regimen of taVNS (25 Hz, 1 mA, 200 µs pulse width) applied for 30 min twice daily significantly reduced median IL-6 levels from 38 to 12 pg ml^−1^ and halved CRP levels compared to sham stimulation (62). Chronic unpredictable mild stress in rodents receiving taVNS reversed anhedonia and hippocampal microglial activation; stimulation upregulated α7nAChR and attenuated JAK2/STAT3 signaling in the prefrontal cortex (63). Similarly, Zucker diabetic fatty rats showed downregulation of proinflammatory P2X7 receptors and synaptic preservation after taVNS, providing a molecular substrate for its antidepressant effect (64).

Collectively, these convergent clinical and preclinical data establish taVNS as a viable, low-risk intervention that simultaneously modulates neural circuits and neuroinflammatory mediators, offering a transdiagnostic platform for improving mood, sleep, and poststroke recovery. Future dose-escalation and biomarker-driven trials are warranted to refine patient selection and stimulus parameters for routine implementation.

### 2.6 Optimization study of stimulation parameters for the anti-inflammatory effect of taVNS

The anti-inflammatory efficacy of taVNS is highly dependent on stimulation parameters, which must be optimized to maximize therapeutic outcomes while ensuring safety and tolerability. This section synthesizes evidence on key parameters—frequency, current intensity, pulse width, session duration, and treatment course—and discusses technological advances enabling personalized dosing (**Table 3**).

**Table 3:** Effects of taVNS stimulation parameter optimization on anti-inflammatory outcomes.

Parameter type	Common scope	Anti-inflammatory effect	Applicable scenarios	Reference
Frequency	1–25 Hz	15 Hz: best anti-inflammatory effect; 100 Hz: strongest brainstem activation	Chronic inflammation, brain disease	Giraudier et al. (67) and Sclocco et al. (89)
Dissociation	0.5–2 mA	High intensity (> 1.5 mA): significant inhibition of proinflammatory factors; individual tolerance adjustment required	Acute inflammation	Owens et al. (68) and Tsaava et al. (70)
Pulse width	250–500 μs	500 μs: enhances nerve activation; 250 μs: reduces skin discomfort	Long-term treatment	Owens et al. (68)
Duration of a single session	15–60 min	≥ 30 min: IL-10 ↑66%; CRP ↓32% (severe COVID-19)	Systemic inflammation	Seitz et al. (7) and Caravaca et al. (42)
Treatment course	4 days–6 months	4 days: improvement in SLE symptoms; 6 months: stabilization of metabolic markers	Maintenance therapy for chronic diseases	Aranow et al. (27) and Sabers et al. (79)

#### 2.6.1 Evolution of taVNS hardware and closed-loop systems

TaVNS hardware has evolved from single-pad electrodes to high-density multielectrode arrays that enable spatially selective activation of the ABVN. Concurrently, closed-loop devices have been developed that integrate real-time physiological feedback, such as HRV or heart rate turbulence indices, to titrate stimulus output dynamically. This approach optimizes cardiovagal gain while minimizing off-target effects (19, 65). For example, dynamic waveform steering that updates polarity, pulse width, and burst pattern every 30–60 s has been shown to double the increase in HRV compared with open-loop paradigms, providing proof of concept for fine autonomic regulation (66).

#### 2.6.2 Key stimulation parameters and their anti-inflammatory effects

Stimulation parameters are typically bounded within 1–25 Hz (with 10–15 Hz most frequently used to augment cardiovagal tone), 0.5–2 mA current amplitude, and 200–500 µs pulse width. This window balances patient tolerability against robust recruitment of the CAP. Low-frequency, high-charge protocols (e.g., 10 Hz, 1.5 mA, 500 µs) preferentially activate CAP-mediated anti-inflammatory cascades, whereas higher-frequency bursts (20–25 Hz) may engage distinct central autonomic nuclei and sympathetic–parasympathetic circuits (67–69).

The dose of stimulation—defined as charge per session (current × pulse width × frequency × duration)—predicts both anti-inflammatory efficacy and tolerability. In severe COVID-19 pneumonia, bilateral taVNS at 15 Hz and 1 mA applied for 60 min reduced CRP by 32% within 24 h and by 80% after 7 days; simultaneously, TNF-α decreased by 58% and IL-10 increased by 66% (7). Switching to 25 Hz while keeping the current constant attenuated this cytokine modulation, suggesting frequency-dependent signaling thresholds (7). Similarly, in rodent models of acute pancreatitis, charge-escalated cervical VNS (20 Hz, 2 mA, 500 µs, 30 min) reduced pancreatic myeloperoxidase (MPO) activity by 55% and TNF-α mRNA by 70%, whereas halving the current reversed these benefits (33, 70).

Excessive charge density can provoke local discomfort or transient bradycardia, underscoring the need for individualized titration. Age, sex hormones, comorbidity burden, and genetic polymorphisms affecting α7nAChR density modulate individual sensitivity (32). Integrating continuous physiological telemetry (HRV, respiration, skin conductance) with machine-learning-driven feedback algorithms now enables real-time parameter optimization and minimization of adverse events (32).

#### 2.6.3 Future directions: toward personalized parameter optimization

Rigorous dose–frequency–response modeling is still required to map the parameter space to molecular and physiological endpoints (71). Future research should leverage machine-learning pipelines trained on large multiomic and neurophysiological datasets to predict optimal stimulus vectors for a given phenotype. Large-scale, phenotype-rich clinical trials will be essential to derive robust, generalizable dosing nomograms for precision taVNS.

### 2.7 Safety and tolerability of taVNS for clinical use

Safety and tolerability of percutaneous auricular vagus nerve stimulation have been systematically evaluated across cardiology, psychiatry, neurology, and endocrinology cohorts. In a multicenter, randomized trial of ST-elevation myocardial infarction (STEMI) patients, 4-day taVNS (25 Hz, 1 mA, 30 min twice daily) lowered in-hospital mortality (4.2% vs. 9.8%, HR = 0.41, 95% CI = 0.19–0.87) and cardiogenic-shock incidence without increasing ventricular arrhythmias (72). Transient, intensity-dependent adverse events included hoarseness, cough, and laryngeal paraesthesia (overall 8%–12%), occurring predominantly when output exceeded 2 mA or pulse width was > 500 µs (73, 74). In major depressive disorder studies, headaches (6%), affective lability (3%), and isolated suicidal-ideation reports (< 0.5%) were confined to the first treatment week and resolved with dose reduction (75). Parkinson’s disease cohorts occasionally exhibited transient facial-motor flicker or gait freezing during 20 Hz bursts, whereas diabetic participants reported mild nausea or bloating that abated when the stimulus duty cycle was shortened (76, 77). Meta-analysis indicates a pooled adverse-event rate of 7.4% (95% CI = 5.2–9.9), with 97% classified as mild and fully reversible; nevertheless, long-term safety in high-risk inflammatory populations remains to be prospectively quantified (78).

The nonsurgical profile of taVNS translates into superior acceptability and adherence. Device-related quality-of-life scores are significantly higher than for implanted VNS (Cohen’s d = 1.24), with avoidance of surgical infection, battery replacement, and cervical fibrosis (22). In refractory depression, self-administered home taVNS achieved 80% adherence at 4 weeks and 68% at 12 weeks; dropout was driven primarily by skin irritation (4%) and time burden (6%), rather than lack of perceived benefit (22). Usability-engineering studies endorse current clip-on electrode designs (System Usability Scale: 84/100), although elderly users report difficulties with cable tangling and impedance checking during long-term use (79). Integration of Bluetooth-guided impedance monitoring, skin-friendly hydrogel pads, and personalized stimulus-titration algorithms improved 12-month adherence to 76% in a recent pilot, underscoring the need for iterative device refinement and adaptive protocols (80). Large-scale, long-term randomized trials powered to evaluate rare adverse events and sustained adherence trajectories remain essential before taVNS can be embedded into chronic-disease management algorithms.

### 2.8 Limitations of current pharmacological anti-inflammatory therapies

The development of taVNS as a therapeutic adjunct must be contextualized within the well-established limitations of conventional anti-inflammatory pharmacotherapies, which span safety, efficacy, accessibility, and economic burden.

First-line agents such as nonsteroidal anti-inflammatory drugs (NSAIDs) and corticosteroids, while effective for symptom control, carry significant risks with chronic use, including gastrointestinal ulceration and bleeding, cardiovascular events, osteoporosis, and heightened susceptibility to infections.

For moderate to severe immune-mediated diseases, biologic agents targeting specific cytokines (e.g., TNF-α, IL-6, IL-17/23) and targeted synthetic disease-modifying antirheumatic drugs (tsDMARDs) have revolutionized care. However, their use is constrained by several major challenges: (1) a substantially increased risk of serious infections, including reactivation of latent tuberculosis (81); (2) high acquisition costs, creating long-term economic burdens for healthcare systems and limiting patient access, especially in resource-limited settings (82); and (3) variable therapeutic responses, with a significant proportion of patients exhibit primary non-response or secondary loss of efficacy over time, necessitating treatment switching (18).

In this context, taVNS represents a non-pharmacological, non-systemic adjunctive strategy. By engaging the body’s endogenous CAP, it may reduce the required dose of concomitant immunosuppressive drugs, potentially mitigating their long-term toxicity and cumulative economic burden, while offering a novel mechanism of action not prone to conventional pharmacological resistance.

## 3 Current challenges and future directions for taVNS

Although the preclinical and clinical data summarized herein are promising, the translation of taVNS into a standardized, widely adopted anti-inflammatory therapy faces several interrelated challenges. Addressing these gaps is pivotal for advancing the field from proof-of-concept studies to precision neuromodulation.

### 3.1 Deepening mechanistic understanding beyond the canonical CAP

While the α7nAChR-mediated CAP provides a fundamental framework, the neuroimmune effects of taVNS are likely more complex. A primary challenge is the delineation of frequency- and circuit-specific mechanisms. Evidence suggests that low-frequency (e.g., 10 Hz) and high-frequency (e.g., 25 Hz) taVNS may engage distinct brainstem nuclei (e.g., nucleus tractus solitarius vs. parabrachial complex) and autonomic outputs, leading to qualitatively different immunomodulatory profiles (69). Future research must employ circuit-level interrogation tools (e.g., conditional knockout models, fiber photometry, optogenetics) in disease-relevant animal models to map the precise neural pathways activated by different taVNS parameters. Furthermore, the role of noncholinergic pathways, including modulation of the hypothalamic–pituitary–adrenal axis, sympathetic noradrenergic signaling to lymphoid organs, and afferent-driven regulation of brain regions such as the locus coeruleus, requires systematic evaluation. Understanding this mechanistic heterogeneity is essential for rationally designing stimulation protocols for specific diseases.

### 3.2 Standardization of stimulation protocols and technological innovation

The field currently suffers from a pronounced lack of standardization, with studies employing highly heterogeneous parameters (frequency, pulse width, intensity, session duration, and treatment course). This heterogeneity obscures dose–response relationships and hinders meta-analyses and clinical replication. A critical future direction is the execution of rigorous, biomarker-stratified, dose-finding studies in human populations. These trials should aim to establish clear correlations between specific stimulation “doses” (charge delivery) and measurable physiological outcomes (e.g., high-frequency heart rate variability increase or a reduction in target cytokines, including IL-6).

Concurrently, technological innovation must focus on developing next-generation, closed-loop taVNS systems. Current open-loop paradigms administer fixed stimulation regardless of the patient’s instantaneous physiological state. Future devices should integrate real-time biosensors (for HRV, skin conductance, or even cytokine levels via wearable assays) with adaptive algorithms that titrate stimulation parameters to maintain a desired neuroimmune state. This bioelectronic “feedback control” promises to maximize efficacy, minimize side effects, and account for individual variability in baseline autonomic tone and disease activity.

### 3.3 Strengthening the clinical evidence base and defining therapeutic niches

Although numerous pilot trials demonstrate efficacy, the overall clinical evidence is characterized by relatively small sample sizes, short-term follow-up, and heterogeneity in sham-control designs (e.g., earlobe stimulation may not be inert). To establish taVNS as a credible therapeutic option, large-scale, multicenter, randomized, double-blind, sham-controlled trials (RCTs) with long-term durability assessments are imperative. These trials should be powered for clinically meaningful endpoints (e.g., ACR50 in RA, endoscopic remission in IBD) and include comprehensive biomarker profiling.

Furthermore, research must better define the optimal therapeutic positioning of taVNS. Key questions include whether it is most effective as a monotherapy for mild disease, a steroid-sparing agent, or an adjunct to biologic therapies in refractory cases. Comparative-effectiveness research against standard pharmacotherapies is needed. Additionally, exploring its utility in conditions with significant inflammatory components but limited treatment options—such as long COVID, fibromyalgia, and aging-related chronic inflammation (inflammaging)—represents a promising future direction.

### 3.4 Pursuing personalized neuromodulation

Acknowledging and addressing interindividual variability is paramount. Factors such as age, sex, anatomical variance in ABVN distribution, baseline vagal tone, genetic polymorphisms in the *CHRNA7* gene (encoding α7nAChR), and concomitant medications can all influence treatment response. The future of taVNS lies in personalization, which involves the development of predictive models using multimodal data (clinical, genetic, electrophysiological, and imaging) to identify *a priori* responders. Treatment protocols could then be tailored, for instance, by using higher current intensities in individuals with low baseline HRV or selecting frequency parameters based on an individual’s disease-specific neuroimmune signature.

In conclusion, overcoming these challenges requires a concerted, interdisciplinary effort bridging neuroscience, immunology, bioengineering, and clinical trial design. By deepening mechanistic insight, standardizing and innovating technology, strengthening clinical evidence, and progressing toward personalized treatment paradigms, taVNS can realize its full potential as a safe, effective, and versatile neuromodulatory therapy for chronic inflammatory diseases.

## 4 Conclusion and future perspectives

This review synthesizes a compelling body of evidence, positioning taVNS as a versatile, noninvasive neuromodulatory tool capable of mitigating inflammation across a remarkably diverse spectrum of diseases. Rather than a mere summary of individual studies, our work provides a novel, integrative perspective by juxtaposing mechanistic insights and clinical outcomes from autoimmune, metabolic, postoperative, and neurological disorders. This cross-disciplinary analysis reveals that the CAP serves as a universal physiological mechanism that can be harnessed therapeutically through a simple auricular intervention.

A central, forward-looking contribution of this review is its critical emphasis on the paramount importance of stimulation parameters. Our synthesis indicates that the anti-inflammatory efficacy of taVNS is not a binary phenomenon but exists on a dose–response continuum. Specifically, low-frequency (e.g., 10–15 Hz), higher-charge-density protocols appear particularly effective for engaging the canonical splenic CAP and suppressing systemic cytokines such as TNF-α and IL-6. This nuanced understanding moves the field beyond the question of whether taVNS works and toward the more critical question of how to optimally deliver it for a given condition.

To translate this promise into clinical reality, we propose concrete directives for future research. First, there is an urgent need for large-scale, rigorously controlled, biomarker-stratified trials to establish evidence-based, condition-specific dosing regimens. Second, mechanistic studies must delineate the frequency-specific neural circuits activated by taVNS to explain why different parameters yield distinct immunomodulatory outcomes. Finally, the field must embrace technological innovation, prioritizing the development of closed-loop systems that titrate stimulation in real-time based on physiological feedback (e.g., heart rate variability), thereby paving the way for truly personalized bioelectronic medicine.

In conclusion, by integrating neuroanatomical, molecular, and clinical evidence, this review establishes taVNS as a potent strategy to recalibrate the brain–body axis. Its capacity to simultaneously modulate innate immunity and autonomic function offers a promising, drug-sparing adjunct for managing chronic inflammation, with the potential to improve long-term outcomes for patients with limited therapeutic options.
